# Thin film resonant metasurface absorbers using patch-based arrays on liquid crystal polymer substrates for centimeter-wave applications

**DOI:** 10.1016/j.heliyon.2024.e35399

**Published:** 2024-07-31

**Authors:** Komsan Kanjanasit, Tanatorn Tantipiriyakul, Changhai Wang

**Affiliations:** a College of Computing, Prince of Songkla University, Phuket Campus, Phuket, 83120, Thailand; b Institute of Sensors, Signals, and Systems, School of Engineering & Physical Sciences, Heriot-Watt University, Edinburgh, EH14 4AS, United Kingdom

**Keywords:** Liquid crystal polymer, LCP, Resonant metasurface absorber, Metasurface, Metafilm

## Abstract

This paper reports the design and development of thin-film resonant absorbers for narrowband and multiband operation in the frequency regions centered at 10 GHz. The structure of the resonant metasurface absorber (RMA) is based on a liquid crystal polymer (LCP) thin-film spacer with a copper patch array on the front surface and un-patterned copper film on the back surface of the LCP film. The design and simulation works were carried out using full-wave analysis of the RMA characteristics. The copper-based periodic patch array acts as a metasurface. The perfect RMA for a given LCP film thickness can be obtained through impedance optimization by adjustment of the dimensions of the lattice periods. The electric and magnetic field distributions were studied. The resonant film absorber based on a 100 μm thick LCP film has an electrical thickness of λ/300 at 10 GHz. The experimental work was conducted using a narrowband RMA prototype consisting of 11×11 cells. The measured result of the resonant absorption is at 10.1 GHz, which is in close agreement with the design frequency of 10 GHz. For multiband functionality, double- and quad-band film resonant absorbers have been designed based on a coplanar supercell utilizing the superposition of the resonance effect. The LCP film-based absorbers have the potential to be used in EM shielding and sensing applications in centimeter-wave applications.

## 1 Introduction

With the advent of wireless communications, radio frequency (RF) technology has become a significant component of the fifth-generation (5G) system, operating over the allocated frequency bands of sub-6 GHz (1-6 GHz) and millimeter-wave (24-40 GHz) ranges. Future sixth-generation (6G) technology is emerging, partially covering the centimeter-wave (7-24 GHz) and sub-terahertz (92-275 GHz) frequency ranges [1]. To interact with RF energy, wave absorbers play a crucial role in electromagnetic (EM) energy-trapping systems. The development of resonant-type absorbers is essential across the entire EM spectrum. One of the unique properties of engineering objects is that they can enable the characteristic of absorption by trapping electromagnetic (EM) energy at specific resonant frequencies or wavelengths based on intrinsic material properties and oriented structure. Metamaterial-based absorbers have been considered for sensing and transmitting RF signals on the Internet of Things (IoT) [2]. The original concept was introduced in the Salisbury screen configuration (radar absorbent material), which was based on a quarter-wavelength thick structure to absorb electromagnetic waves [3]. The main focus of designing RMA has been on achieving a thin-profile structure. By utilizing a frequency-selective surface (FSS) or resistive surface, the RMA structure can be reduced to less than a quarter wavelength [4]. The trapped-mode effect in RMA, associated with a plasmonic dark mode, has been addressed through the interaction of coupled resonators in a coplanar structure operating in the microwave region [5][6]. This represents a key element in enabling a zero-thick absorption mechanism, although the resonant absorption may be imperfect. To achieve a perfect RMA, a configuration based on a double-sided metamaterial combining dissimilar periodic surfaces at the microwave frequency [7]. Each of these periodic surfaces provided electric and magnetic responses, ensuring a matching surface impedance. This concept demonstrated the importance of artificial structures as the essential elements in the design of perfect RMAs.

On the other hand, the high-impedance surface (HIS) configuration also represents a powerful mechanism for creating a perfect RMA structure by incorporating a periodic resonator array on a grounded dielectric slab [8]. The EM activity in this configuration relies on the interference between the networked resonant elements and the ground surface across the thickness of the dielectric slab and the frequency-dependent properties of the effective permittivity ϵ(ω) and permeability μ(ω) have been engineered to be dispersive to enhance resonant absorption [7][9]. By achieving zero transmission, the HIS configuration provides anti-reflection capabilities by controlling the wave impedance to match that of the intrinsic absorber impedance. However, the criticality of the ultrathin absorber thickness requires accurate analysis using a structural model that accounts for the near-field interaction in the two-layer structure [10]. The presence of lossy media further complicates the analysis due to the complex terms of the electric permittivity and magnetic permeability, which affect the impedance response. Several studies have utilized HIS-based EM wave absorbers to explore absorbing configurations across various EM frequency regions, including microwave, millimeter-wave, terahertz, infrared, and visible light spectra [11], [12], [13], [14], [15], [16], [17], [18], [19], [20], [21].

In various applications, microwave absorbers were initially utilized for radar cross-section (RCS) reduction in stealth technology within the defense sector [22]. Other examples include integrating millimeter-wave absorbers into pyroelectric detector sensors to enhance sensing performance [23] and developing solar energy harvesting applications for high-efficiency conversion [24]. Resonant absorbers exhibit several features, such as broadband response, multiband operation, arbitrary polarization, and wide-angle sensitivity. These characteristics make them suitable for applications such as wearable wireless healthcare devices and microwave energy harvesting. They are also used for microwave-band shielding, providing field protection [25], and as sensors in industrial, medical, and agricultural products [26]. Resonant absorbers are incorporated into sensor module assemblies to interact with the incident EM waves. However, microwave-band resonant absorbers tend to have large dimensions due to the wavelengths involved in their physical design. Conventional rigid dielectric substrates have limitations for achieving ultrathin absorbers. To synthesize lossy mediums, specific substances are added to extend the configuration, and the sensing performance is a concern when operating in high-frequency applications.

With the advancement of novel polymeric materials, liquid crystal polymer (LCP) stands out as a thermoplastic polymer material with attractive attributes in electromagnetic, mechanical, electrical, and chemical properties [27]. LCP substrates have gained attention in designing high-frequency devices and applications due to their good thermal stability, low water absorption, low loss, and high efficiency [28]. In recent years, LCP thin films have been exploited in 5G applications spanning microwave, millimeter-wave, and terahertz bands [29]. The electrical properties of LCP have been reported at high frequencies (up to 110 GHz) to exhibit a dielectric constant (relative permittivity) ranging from 2.9 to 3.2 and a dissipation factor (loss tangent) slightly varying from 0.002 to 0.005. These characteristics make LCP an excellent material for applications across a wide range of electromagnetic frequencies [30][31]. The utilization of LCP substrates has contributed to various areas such as micro-electro-mechanical systems (MEMS), antennas and metamaterials, sensors, and microfluidics. High-frequency applications hold significant potential in modern technologies, including multilayer circuits and circuit packaging, flexible and wearable electronics, circuit devices and interconnections, as well as biomedical and implantable devices. Designs of RF devices using LCP substrates have been proposed, such as microwave filters [32], RF-MEMS switches [33], and millimeter-wave antennas [34]. Additionally, the low cost of LCP substrates provides an advantage in terms of cost-effective manufacturing of RF-based electronic products.

This paper reports the studies of the LCP film substrate-based ultrathin RMAs operating in the centimeter-wave range. LCP film is used in the design of narrowband and multiband film-resonant absorbers based on a fundamental HIS configuration. The trapped-mode mechanism in film-resonant absorbers is illustrated through electromagnetic simulation and analysis. The absorption characteristics have been studied and realized by adjusting the lattice period parameters and film thicknesses using commercially available LCP substrates. We investigated wave-impedance matching methods to achieve perfect absorption at the targeted frequencies. The narrowband RMA is experimentally verified and compared with the results of numerical simulation and analysis.

## 2 LCP-based film-resonant absorber

### 2.1 LCP material

The LCP dielectric thin film materials are commercially available with thicknesses ranging from 25 to 100 μm. As flexible substrates for circuit construction, the LCP films are laminated with a standard copper cladding of 18 μm of thickness. The electrical conductivity (*σ*) of the LCP films is 5.8 × 10^7^ S/m. Table 1 provides the electrical, mechanical, and thermal properties of an LCP film at the frequency of 10 GHz [35]. The LCP film possesses unique properties that make it suitable for high-frequency applications, including a low dielectric constant and a low loss dissipation factor in the GHz frequency bands. Its excellent mechanical and thermal characteristics make it beneficial for system-on-a-package (SoP) technology using modern assembly methods. In this study, commercial LCP films (Ultralam 3850, Rogers) with three standard thicknesses of 100 μm, 50 μm, and 25 μm were used to investigate the film-resonant absorbers. For the targeted frequency of 10 GHz, the thickness-to-wavelength factors are 0.0033, 0.0017, and 0.0008 for the respective LCP thicknesses. These values indicate the extremely thin structure of the film-resonant metamaterial absorbers.

**Table 1:** Properties of LCP dielectric film (Ultralam 3850, Rogers).

Property	Value	Unit
Dielectric constant (10 GHz)	2.9	-
Dissipation factor (10 GHz)	0.0025	-
Surface resistivity	1 × 10^16^	Ω
Volume resistivity	1 × 10^18^	Ω*cm*
Tensile strength	200	MPa
Young's modulus	2255	MPa
Melting temperature	315	°C
Coefficient of thermal expansion (CTE)	17	ppm/°C
Water absorption (23 °C, 24 hrs)	0.04	%

### 2.2 Absorber structure and design

**Figure 1:**
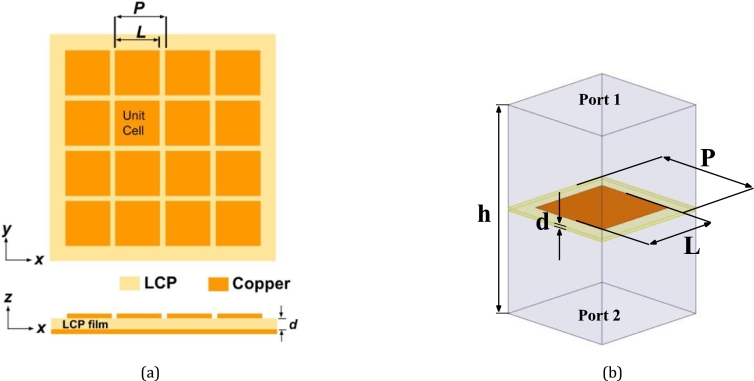
The schematic layout of (a) the LCP film-resonant absorber and (b) the unit-cell structure for electromagnetic simulation: *P* is a 2D square periodic space, *L* is the length of a square patch, *d* is the thickness of the LCP dielectric, and *h* is the distance between two ports of the periodic boundary environment.

Fig. 1(a) illustrates a section of the RMA structure utilizing the LCP film. The schematic layout is presented in both the top (x-y) and cross-section (x-z) views featuring a double-sided configuration. The LCP film is between a square copper patch array acting as a capacitive-type frequency-selective surface (FSS) on the top (front) surface. The bottom copper layer is the ground plane. The choice of patch-based elements in the design is based on their fundamental resonator structure and a higher degree of geometrical symmetry. The absorber configuration can be viewed as an ultrathin cavity, with the two surfaces forming a parallel structure. The analysis in [8] provides a detailed representation of the operation of the structure, and field interference is the dominant effect due to the extremely small separation between the FSS and the ground plane. In this design, two physical parameters, namely the length (*L*) of a square patch and the period (*P*) of the square lattice, are determined to achieve resonant absorption at the targeted frequency. The patch element functions as a resonator following the principle of the patch antenna theory, where the length *L* corresponds to approximately half guided wavelength (∼0.5$\lambda_{g}$) [36]. The square patch has dimensions of 8.5 × 8.5 $mm^{2}$ determined and optimized to resonate at 10.0 GHz based on the LCP dielectric properties, $\lambda_{g}=\lambda_{o}/\sqrt{\epsilon_{r}}$ where $\lambda_{o}$ is the free space wavelength for 10 GHz of frequency. The periodic surface serves the purpose of interacting with the incoming field, thereby enhancing the performance of perfect absorption through impedance matching. In this context, the lattice period (*P*) is a significant factor in controlling the wave impedance, and the optimization of impedance matching is achieved through a parametric study.

Fig. 1(b) illustrates the unit cell structure of the LCP film RMA based on the Floquet theorem. The unit cell is located within a periodic boundary. Two ports are positioned on the top and bottom walls of the boundary box, allowing for plane-wave excitation to obtain the EM scattering response. This model effectively represents an infinite periodic planar structure. The reflection response is analyzed to investigate the impedance reciprocity in the interfacial media, with the EM interaction expressed in terms of the reflection magnitude $S_{11}$[37]

(1) $$|S_{11}|=\sqrt{\frac{{\left(Re\{Z_{s}\}-Z_{w}\right)}^{2}+{\left(ImZ_{s}\right)}^{2}}{{\left(Re\{Z_{s}\}+Z_{w}\right)}^{2}+{\left(ImZ_{s}\right)}^{2}}}$$

where $Z_{S}$ is denoted as the input surface impedance, while $Z_{W}$ is represented by the wave impedance (377 Ω). To fulfill the resonance condition, the surface impedance should have only a real part, and its value should be equal to the wave impedance in the non-reflecting boundary condition at the media interface. In a mismatched condition, the impedance effect becomes dominant and significantly affects the magnitude of the resonant absorption.

## 3 Simulation of RMAs

The characteristics of the resonant absorber structures were conducted using the ANSYS HFSS software based on the finite element method. The investigation was carried out under normal incidence to obtain the reflection ($S_{11}$) and transmission ($S_{21}$) parameters. The field incidence was applied to the periodic patch surface, and the scattering parameters were calculated across a frequency range to analyze the electromagnetic interaction. The absorbance characteristics, A(ω), were determined as a frequency-dependent quantity expressed as

(2) $$A(\omega )=1-|S_{11}(\omega ){|}^{2}-|S_{21}(\omega ){|}^{2}$$

In terms of the absorber structure, the transmission ($S_{21}$) value is close to zero, indicating that no energy passes through the ground surface at the bottom (rear) surface of the LCP film. In the case of achieving total absorption, a zero-reflection magnitude is obtained by employing the perfect impedance matching method.

### 3.1 Resonance effect in absorption

**Figure 2:**
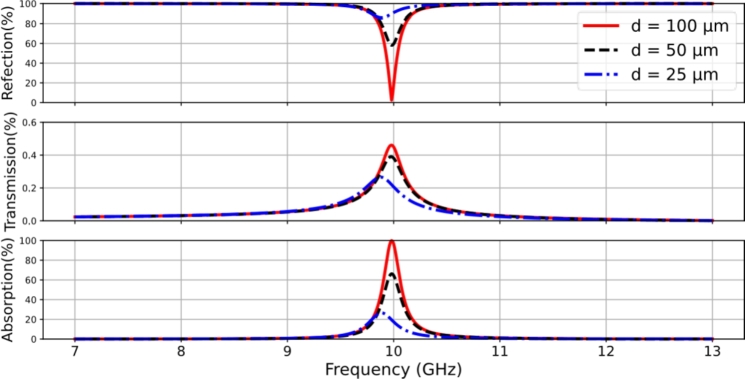
Simulated results of the characteristics of reflection, transmission, and absorption for the LCP film thicknesses of 100 μm, 50 μm, and 25 μm as a function of frequency.

Fig. 2 shows the reflection, transmission, and absorption characteristics of the LCP-based thin film absorbers over the frequency range of 7-13 GHz. Different LCP thicknesses were investigated while keeping the patch dimension and lattice period constant in a unit cell. The resonant profiles indicate the appearance of a near-zero reflection dip at the target frequency of 10 GHz. Small transmission peaks of around 0.6% are observed through the ground surface due to the induced electromagnetic field by the cavity effect of constructive interference (the ground surface behaves as a mirror of the patch array). The resonant absorptions are evident with a peak of approximately 100% (unitary peak) for the case of *d* = 100 μm, while for LCP thicknesses of *d* = 50 μm and *d* = 25 μm, the absorption drops to 66.3% and 22.6%, respectively. The results demonstrate that the resonance process facilitates absorption at the desired narrowband frequency. The obtained quality factor for perfect resonant absorption is 52.6. The magnitudes of the reflection peaks are influenced by the impedance matching condition at the media interface between free space and the RMA surface. Good impedance matching is achieved at *d* = 100 μm, where the characteristic impedance of the incident surface is equal to the wave impedance. This finding reveals that the characteristic impedance of a grounded LCP film depends on its thickness, as indicated in Reference [7]. Therefore, this investigation highlights the impedance effect in the resonant absorption structure, which is associated with the thickness constraint of the film material.

### 3.2 Effect on surface impedance

**Figure 3:**
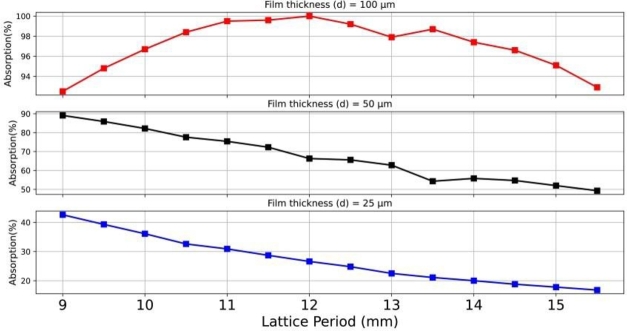
Responses of the resonant peaks of the absorption magnitude for the commercially available LCP thicknesses of 100 μm, 50 μm, and 25 μm with varying lattice periods.

To control the reflecting surface impedance, the designs of the square lattice are considered for impedance matching optimization. The perfect condition of the reflecting surface requires excellent matching to the wave impedance (377 Ω). A parametric study was conducted to vary the square lattice period (parameter *P*) within the range of 9.0-15.5 mm (0.3-0.52$\lambda_{o}$). Meanwhile, the patch dimensions were kept constant at 8.5 × 8.5 $mm^{2}$ designed for 10 GHz operation. Fig. 3 presents plots of the peak resonant absorption. It is observed that the absorption peak approaches unity (100%) when varying the lattice period for the case of *d* = 100 μm. However, for the case of *d* = 50 μm and *d* = 25 μm, the peak responses are imperfect, i.e., not close unity, only achievable in the range of 89.1% to 49.3% and 42.6% to 16.8%, respectively. The use of a 100-μm-thick film demonstrates total absorption achieved at the lattice period of *P* = 12.0 mm, corresponding to a period-to-wavelength factor of 0.4, which ensures perfect impedance matching. Hence, the LCP film thickness of 100 μm with the optimal period holds the potential for achieving perfect resonant absorption. The optimization of the periodic surface enhances the absorption capability by tuning the surface impedance.

**Figure 4:**
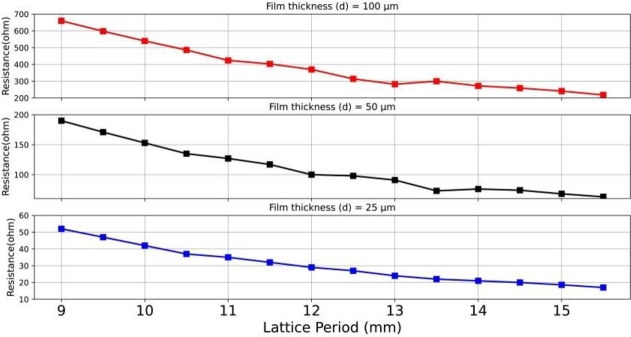
Responses of the peaks of surface resistance (the real part of input impedance) for the LCP-based absorbers with varying thicknesses of 100 μm, 50 μm, and 25 μm with varying lattice periods.

Fig. 4 illustrates the three study cases with different thicknesses of 100 μm, 50 μm, and 25 μm in terms of the resistance response of the surface impedance at the resonant frequency of absorption, with varying lattice periods. In the case of *d* = 100 μm, the response indicates a gradual decrease in the surface resistance at the resonant absorption, ranging from 660 Ω to 218 Ω as the lattice period parameter was varied between 9 mm and 15.5 mm. It demonstrates that a matched resistance of approximately 377 Ω is achieved at a lattice period parameter of *P* = 12 mm, providing a perfect matching condition. For the cases of *d* = 50 μm and *d* = 25 μm, the imperfect resonant absorptions result in resistance ranges of 190 Ω to 63 Ω and 52 Ω to 17 Ω, respectively. It is crucial to note that the unmatched impedance under these film-thickness conditions is due to the influence of higher-order Floquet modes (evanescent modes) on capacitance and inductance [38]. Therefore, the results highlight the importance of the optimal lattice period as a significant factor in tailoring the surface impedance for achieving perfect matching.

### 3.3 Characteristics of surface current and EM field

**Figure 5:**
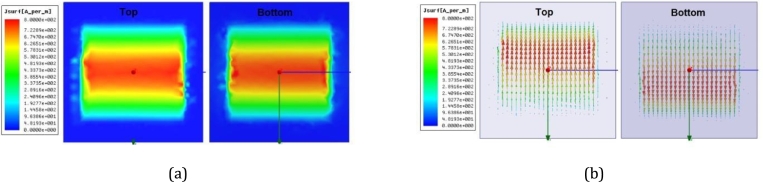
Snapshots of the surface current distribution in the unit cell depicting the magnitude (a) and vector (b).

To better understand the absorption mechanism, an EM field simulation is presented to illustrate the surface current distribution on both the top and bottom surfaces at 10 GHz. Fig. 5 displays the magnitude and vector features of the surface current profile. It reveals that the response of the fundamental-mode resonance occurs on the actual patch element (top surface), while a virtual patch object is formed through the EM mirror effect on the ground surface (bottom surface). The surface current exhibits a similar profile on both surfaces, but the vector configuration is out of phase. This arrangement demonstrates the formation of anti-phase parallel surface currents on the double-sided film substrate. This unique distribution is associated with the field-coupling resonance in the absorption mode [4][5][36].

**Figure 6:**
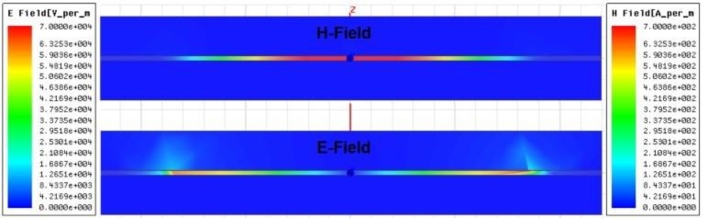
Snapshots of the electric (*E*) and magnetic (*H*) fields in the cross-section view of the unit cell.

Fig. 6 shows the characteristics of the electric (*E*) and magnetic (*H*) field distributions in the x-z plane at the resonant absorption frequency of 10 GHz. The electric field is observed to emerge at the edges of the patch element due to the charge interaction between the actual and virtual patches and the neighboring unit cells within the periodic network. On the other hand, the magnetic field exhibits significant strength in the inter-surface region (LCP dielectric space) as a result of the orientation of the anti-phase surface current. This manifestation of the EM field effect signifies the absorption mechanism. The LCP thin film demonstrates the capability to function as a narrowband resonant absorber with trap-mode behavior when subjected to the targeted impinging EM field.

### 3.4 RMA verification

**Figure 7:**
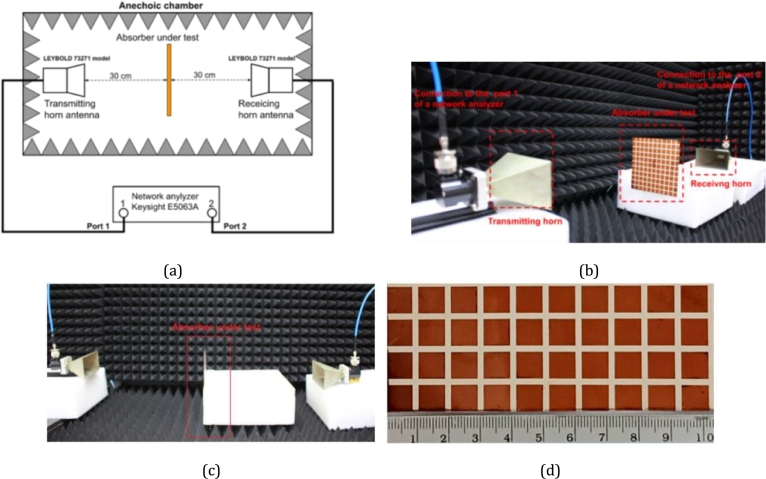
The measurement setup for the characterization of the fabricated LCP film resonant absorber, (a) schematic diagram, and (b) and (c) views of the actual setup in the anechoic chamber, and (d) phototype of the RMA.

A fabricated RMA prototype was produced using a microfabrication method and a high-resolution photoresist material. For the sample prototype, the RMA design with a lattice period of 12.0 mm was chosen, using a commercial 100-μm-thick LCP-film substrate with double-clad copper (Ultralam 3850, Rogers). The RMA prototype consisted of 11 × 11 patch cells, resulting in a total dimension of 252 × 252 $mm^{2}$. Fig. 7 shows the microwave measurement setup, where a line-of-sight distance was employed to characterize the scattering properties under normal incidence, as detailed in Reference [39]. The setup was made using a network analyzer (Keysight E5063A model) and standard horn antennas (LEYBOLD 73271 model) within an anechoic chamber. The transmitting and receiving horn antennas served as wave ports in the simulation and were connected to Port 1 and Port 2 of the network analyzer, respectively. To support the horn antennas and the RMA in the setup, a foam material with a dielectric constant close to that of air was utilized. The fabricated RMA prototype was positioned at a distance of 30.0 cm (10*λ* at 10 GHz) from the horn antennas, corresponding to the far-field distance. This distance ensured that a plane wave for field propagation was excited by the horn antenna.

**Figure 8:**
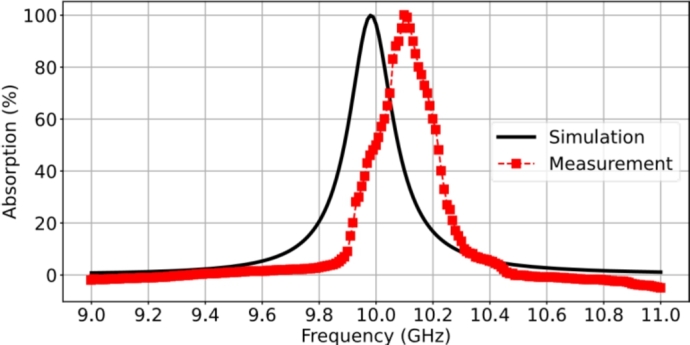
The measured result of the fabricated LCP film-resonant absorber and the simulation results.

The measurement procedure involved two steps: one with a blank copper surface and the other with the actual RMA. The reflection and transmission responses were obtained within the frequency band of 9-11 GHz. Firstly, the blank copper sheet, which served as a total reflective surface and was the same size as the RMA, was used to obtain the background response for normalization. Secondly, the reflection and transmission coefficients of the actual RMA were measured and then normalized using the response of the blank copper sheet. The normalized reflection and transmission responses were calculated to determine the absorption. The key result of the measured reflection response exhibited a sharp dip close to zero at 10.1 GHz. Fig. 8 shows the results of the absorption from the measurement and simulation. The agreement between the measurement and the simulation is excellent, only showing a small frequency shift of 100 MHz, which is attributed to the imperfections in the fabrication process, such as the effect of the etching process on the actual dimensions of the patches. The measured response indicates a resonant frequency of absorption and a peak value of unity.

## 4 Multiband film-resonant absorbers

In this section, we describe the design and simulation of resonant meta-surface absorbers for multiband applications. The 2-D periodic structures were designed using supercells, which can produce multiband resonant absorption responses. As discussed in the previous section, the 2-D uniform patch-based RMA offers narrowband resonant absorption. However, by incorporating patches of different dimensions in a supercell, LCP-based double-band and quad-band RMAs have been designed.

### 4.1 Resonant absorbers of different patch dimensions

The operating frequencies of individual resonant absorbers were investigated to determine their resonant frequencies. Five different dimensions of periodic patch-type resonators are studied, and the film-resonant frequency responses were investigated by varying the patch dimensions. The length (*L*) of the square patch in each array is selected as 7.5 mm, 8.0 mm, 8.5 mm, 9.0 mm, and 9.5 mm while maintaining a lattice period (*P*) of 12.0 mm.

**Figure 9:**
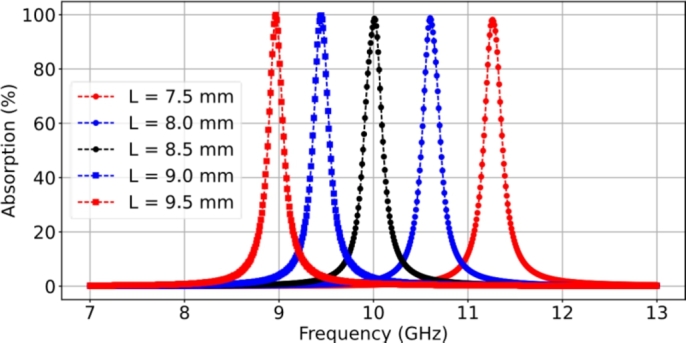
Simulated results of the characteristics of the absorption for a variation of the square patch dimensions (*L*) consisting of 7.5 mm, 8.0 mm, 8.5 mm, 9.0 mm, and 9.5 mm.

Fig. 9 displays the plots of the simulated absorption responses as a function of frequency over the range of 7-13 GHz. The resonant absorption occurs at frequencies of 11.26 GHz, 10.55 GHz, 10.1 GHz, 9.45 GHz, and 8.95 GHz, respectively. These resonant frequencies depend on the patch lengths (*L*) and are approximately half-guided wavelengths, satisfying the resonance condition. Perfect impedance matching with near unity peaks is achieved in all cases of resonant absorption.

### 4.2 Double-band resonant absorbers

**Figure 10:**
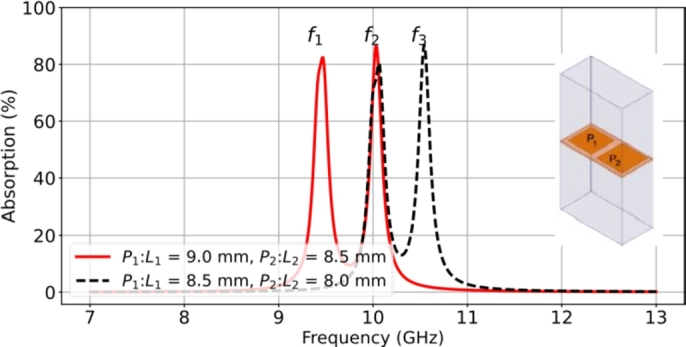
Simulated results of the characteristics of the double-band resonant absorber for supercell designs consisting of two different square patches (*P*_1_ and *P*_2_).

A periodic arrangement of the supercell is formed using two patches in a supercell to create a double-band resonant absorber. The lattice dimensions are 12.0 × 24.0 $mm^{2}$, corresponding to the space of two periods in a single patch-based cell. The supercell consists of two different sizes of patch elements. In order to achieve a double-band response, two patches with different sizes are arranged in a subarray connected with the intrinsic modes of the resonant patch elements. Two cases of the supercells are studied, denoted as Case I and Case II, with different patch dimensions placed on a 100 μm thick LCP film. In Case I, the lengths of the square patches are $L_{1}$ = 9.0 mm and $L_{2}$ = 8.5 mm, while in Case II, the lengths of the square patches are $L_{1}$ = 8.5 mm and $L_{2}$ = 8.0 mm. It is observed that the common patch size is 8.5 × 8.5 $mm^{2}$. The supercell was simulated to demonstrate the double-band response, as shown in Fig. 10.

The simulation results show double-band resonant absorption in both cases. The resulting frequency peaks correspond to those of the RMAs consisting of the individual patches. In Case I, the resonant frequencies occur at $f_{1}$ = 9.46 GHz (peak 82.3%) and $f_{2}$ = 10.03 GHz (peak 86.4%), while in Case II, the resonant frequencies are at $f_{2}$ = 10.03 GHz (peak 80.4%) and $f_{3}$ = 10.56 GHz (peak 86.9%). It is noted that the same resonant absorption occurs at the frequency $f_{2}$ due to the common patch size in both cases. The resistance result of the interfacial surface impedance is unmatched, resulting in 129 Ω and 150 Ω in Case I. Case II is also the same condition, resulting in 94.2 Ω and 91.9 Ω.

**Figure 11:**
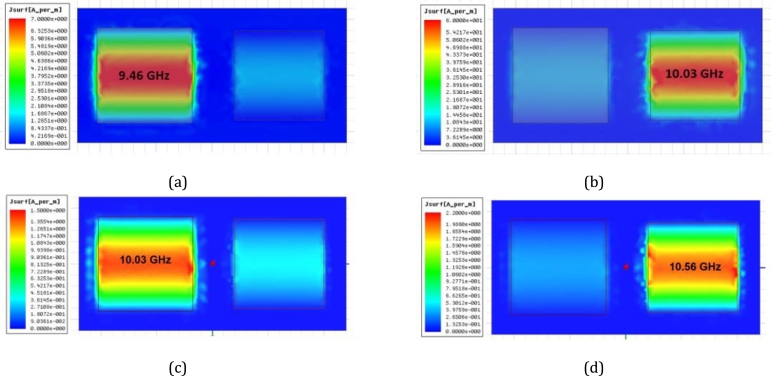
Snapshots of the surface current magnitude in the unit cell of the double-band absorber, for Case I at (a) *f*_1_ = 9.46 GHz and (b) *f*_2_ = 10.03 GHz; Case II at (c) *f*_2_ = 10.03 GHz and (b) *f*_3_ = 10.56 GHz.

The impedance optimization was modified to decrease the lattice period to 9.5 × 19.0 $mm^{2}$. This led to an improvement in the resonant absorption with peaks of 96.0% (309 Ω) and 92.5% (233 Ω) in Case I. Case II was also examined, resulting in peaks of 92.7% (325 Ω) and 96.3% (266 Ω). The optimization of the lattice period was limited by the gap between patches. To understand the EM activity in the supercell structure, the surface current distribution was simulated to illustrate the resonance condition. Fig. 11 presents the surface current profiles both Case I and Case II. The resulting current behavior independently controls the two absorption peaks. The intrinsic resonant frequencies $f_{2}$ and $f_{3}$ are based on the resonance properties of patch $P_{1}$, patch $P_{2}$, and patch $P_{3}$, respectively.

### 4.3 Quad-band resonant absorbers

**Figure 12:**
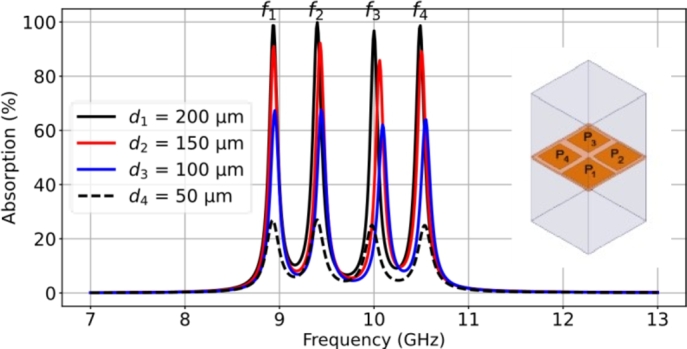
Simulation results of the characteristics of the quad-band resonant absorption for four different LCP film thicknesses consisting of four different square patches (*P*_1_, *P*_2_, *P*_3_, and *P*_4_).

**Figure 13:**
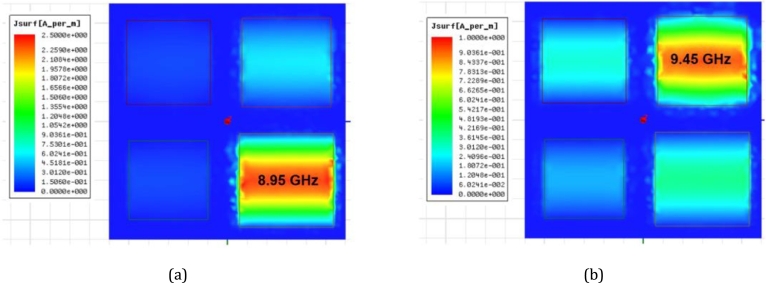

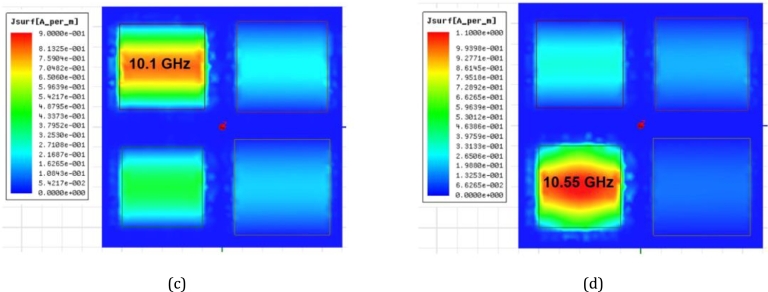
Snapshots of the surface current magnitude in the unit cell of the quad-band absorber, (a) 8.95 GHz, (b) 9.45 GHz, (c) 10.1 GHz, and (d) 10.55 GHz.

**Figure 14:**
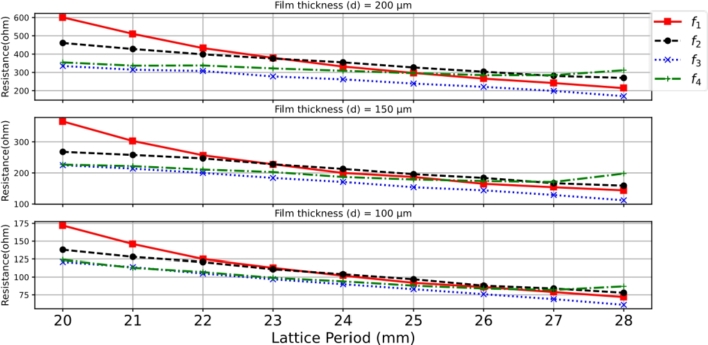
The surface resistance peaks of the quad-band resonant absorber at *f*_1_, *f*_2_, *f*_3_, and *f*_4_ for the LCP thicknesses of 200 μm, 150 μm, and 100 μm.

The square supercell of the quad-band resonant absorber was formed by a 2 × 2 subarray, with a size of 24.0 × 24.0 $mm^{2}$. It contains four patch elements of different sizes, namely patch $P_{1}$ = 9.5 × 9.5 $mm^{2}$, $P_{2}$ = 9.0 × 9.0 $mm^{2}$, $P_{3}$ = 8.5 × 8.5 $mm^{2}$, and $P_{4}$ = 8.0 × 8.0 $mm^{2}$. These patches were arranged within the supercell space and repeated on the LCP film substrate. The supercell was simulated to analyze the response of the resonant absorption, as shown in Fig. 12. The simulations were carried out for the variation of the LCP film thickness, 50 μm, 100 μm, 150 μm, and 200 μm. The resonant absorptions occur at frequencies $f_{1}$ ≈ 8.94 GHz, $f_{2}$ ≈ 9.42 GHz, $f_{3}$ ≈ 10.02 GHz, and $f_{4}$ ≈ 10.52 GHz in the centimeter-wave range. These resonant frequencies correspond to those of the RMAs consisting of the individual patches in periodic arrays. Considering the case of the 100-μm-thick LCP film, the resistance decreases for the four resonant frequencies, 102 Ω, 104 Ω, 90 Ω, and 94 Ω, resulting in imperfect matching impedance. As a result, the absorption peaks are around 67.4%, 67.6%, 62.1%, and 64.0%, respectively. To enhance the absorption peaks, the LCP film thickness was increased to optimize the surface impedance of the interfacial absorber. It is observed that a 200-μm-thick film substrate provides an improvement in achieving near-perfect absorption. The quad-band absorption results in peak values of 90.7% (332 Ω) at $f_{1}$, 99.9% (355 Ω) at $f_{2}$, 96.8% (262 Ω) at $f_{3}$, and 98.7% (309 Ω) at $f_{4}$ respectively. In the other cases of the varied thickness, the impedance is low and unmatched. Fig. 13 shows the characteristics of the surface current profiles at the resonant frequencies: $f_{1}$ = 8.95 GHz, $f_{2}$ = 9.45 GHz, $f_{3}$ = 10.1 GHz, and $f_{4}$ = 10.55 GHz. The simulation was to understand the electromagnetic activity of the multiband-mode operation. The results show that the superposition of the resonance effects exhibits resonant absorption dominated by the individual patch within the supercell. Fig. 14 shows the resistance response for the four resonant frequencies ($f_{1}$, $f_{2}$, $f_{3}$, and $f_{4}$) as the period (*P*) for the LCP film thicknesses of 200 μm, 150 μm, and 100 μm. The dimensions of the square supercell were varied between 20 × 20 $mm^{2}$ and 28 × 28 $mm^{2}$. The results indicate that good impedance matching can be achieved with a lattice period (*P*) around 22 ∼ 24 mm, specifically in the case of the LCP film thickness of 200 μm. Thus, it is evident that both the film thickness and lattice period are significant factors in optimizing the impedance to achieve a near-perfect quad-band resonant absorber.

### 4.4 Verification and discussion

**Figure 15:**
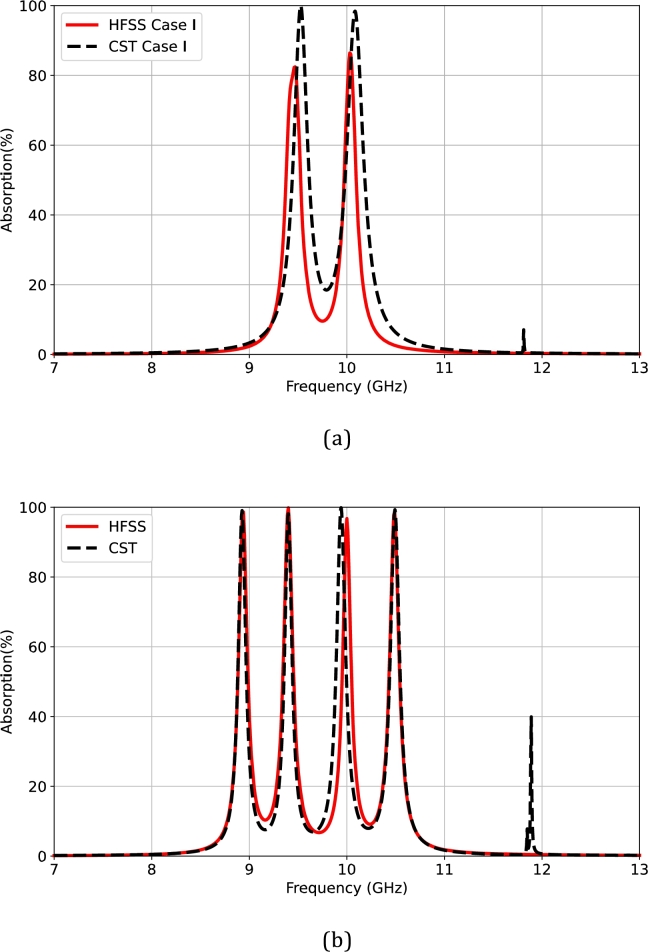
Comparative analysis in the design of (a) the double-band and (b) quad-band resonant absorption between the HFSS and CST software.

A comparative numerical analysis is performed to examine the characteristics of the multiband RMA designs. The full-wave simulation is conducted using a frequency-domain solver of the 3D EM analysis in CST Studio Suite software. To verify the multiband absorption, the quad-band RMA design, utilizing a 200-μm-thick LCP material, has been simulated and compared with results from a previous HFSS-based simulation. Fig. 15 presents the comparative results, demonstrating correlation in terms of the frequencies for the double- and quad-band RMAs with the two and four peaks of the resonant absorption. A small shift is observed around the resonant frequency.

**Figure 16:**
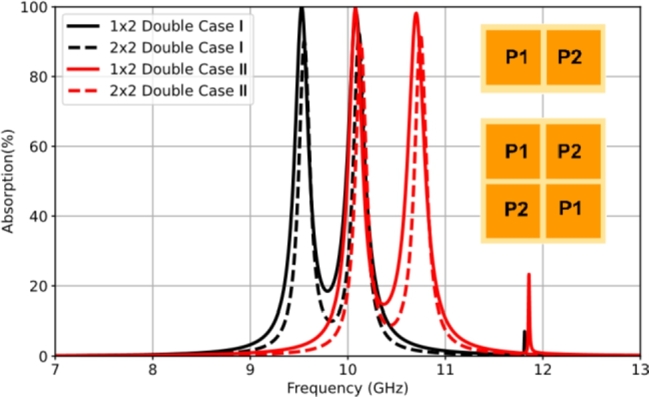
A comparative simulation of the double-band resonant absorption in the 2 × 2 diagonal orientation between Case I and Case II.

In the case of the dual-band RMA, a design topology based on a 2 × 2 diagonal orientation was implemented to investigate the frequency response of resonant absorption. Fig. 16 illustrates the characteristics of the dual-band absorption for two cases (Case I and Case II) using multiplexing patch elements in a supercell. With the CST-based simulation, the frequency responses are validated at 9.46 GHz and 10.03 GHz in Case I. In contrast, the operating frequencies in Case II are confirmed at 10.03 GHz and 10.56 GHz. This study provides a clear understanding of the effects of diagonal orientation.

**Figure 17:**
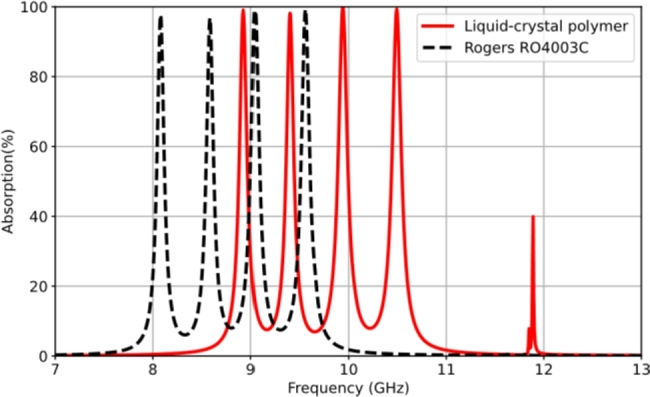
Simulation results in the case of the quad-band resonant absorption using RO4003C (Rogers PCB) material compared with the LCP dielectric.

The investigation into using a different material property is reconsidered with a high-frequency circuit material, specifically RO4003C (Rogers PCB) [40]. This material is a ceramic-based structure that benefits from compatibility with printed circuit board fabrication processes. It possesses a dielectric constant of 3.38 and a loss tangent of 0.0027. The volume resistivity is 1.7 × 10^16^ Ω cm, and the surface resistivity is 4.2 × 10^15^ Ω. The dielectric host of the quadband RMA configuration, maintaining the same design parameters, is replaced with this material to examine the operating frequencies. Fig. 17 illustrates the characteristics of the quadband resonant absorption at 8.08 GHz (97.7%), 8.58 GHz (96.8%), 9.05 GHz (99.01%), and 9.56 GHz (99.3%). This simulation provides a perspective of the effects of the varied relative permittivity.

Table 2 shows a comparison of existing resonant absorber research with related designs. The recently proposed absorber structures in these works correspond to periodic surfaces and planar dielectric-hosted materials with complex unit-cell patterns. The comparative elements include the type of dielectric material, host thickness, operating frequency, EM applications, and the peaks of absorptivity. The classification into millimeter (mm) wave and centimeter (cm) wave ranges refers to wireless communication technology. FR4 is frequently used as a lossy dielectric due to its cost-effectiveness. To take into account multiple research studies, the Eccosorb® SF product, a resonant microwave absorber from Laird, is researched and developed as a narrowband resonant absorber. The thickness varies from 1.27 mm to 4.7 mm, depending on the frequency, which ranges from 1 to 26 GHz [41]. This proposed work provides an ultrathin resonant absorber where multiplexing independent resonance conditions can design the adjacent target frequency.

**Table 2:** Comparison of related RMA designs with existing works.

Ref.	Dielectric	Dielectric	Operating	Applications	Absorptivity
	type	thickness	frequency		
[15] 2023	FR4	2.0 mm	Five-band	5G mm-wave	92.9∼99.3%
			20∼26 GHz		

[16] 2024	FR4	0.34 mm	Dual-band	5G mm-wave	∼99%
			28 GHz and 39 GHz		

[17] 2022	FR4	1.6 mm	Multiband	5G mm-wave	90∼100%
			22∼36 GHz		

[18] 2020	FR4	2.0 mm	Wideband	cm-wave	∼95%
			8.3∼11.3 GHz	(X-band)	

[19] 2019	FR4	1.4 mm	Six-band	cm-wave	95.4∼99.2%
			5∼14 GHz	(C, X, Ku-band)	

[20] 2019	FR4	1.0 mm	Triple-band	cm-wave	96.36∼99.95%
			9∼15 GHz	(X, Ku-band)	

[21] 2019	Rogers	1.5 mm	Dual-band	cm-wave	∼99%
	RO3003		2.4 GHz and 5 GHz	(WiFi)	

This work	LCP	0.1∼0.2 mm	Single 10 GHz	cm-wave	98.2∼99.9%
			Double & Quad-band	(X-band)	
			over 9∼11 GHz		

## 5 Conclusion

The LCP film-based resonant meta-surface absorbers have been successfully demonstrated in the centimeter-wave region around 10 GHz. The analysis of the RMA configurations based on full-wave simulations has provided significant insights into the square-patch-based periodic surface on the grounded LCP substrate. A narrowband perfect RMA structure has been achieved using a 100-μm-thick LCP film with an electrical thickness of *λ*/300. Furthermore, multiband RMA structures based on a coplanar supercell have been investigated, allowing the design of double- and quad-band absorption responses using multi-sized patch elements. The multiband absorption characteristics are associated with the superposition of resonances. The results of the study indicate that the LCP film thickness and the optimal lattice period of the patch array are the key factors for manipulating the surface impedance to achieve a well-matched condition. The resonant absorption in the double-sided structure is influenced by EM perturbation, which results in a localized surface current. The anti-phase coupling of surface currents with the magnetic field response in the LCP film reveals the trap-mode effects. The LCP dielectric film behaves as a trap-mode artificial magnetic conductor exhibiting concentrated magnetic field lines. These LCP-based resonant absorbers have potential applications in next-generation smart communication systems benefiting from the advancements in thin film technology.

## Funding

This work is partially funded by College of Computing, Prince of Songkla University Phuket Campus, Thailand (Project numbers COC6602073N and COC6604012S).

## CRediT authorship contribution statement

**Komsan Kanjanasit:** Writing – original draft, Visualization, Validation, Methodology, Formal analysis, Conceptualization. **Tanatorn Tantipiriyakul:** Validation, Methodology, Conceptualization. **Changhai Wang:** Writing – review & editing, Validation.

## Declaration of Competing Interest

The authors declare the following financial interests/personal relationships which may be considered as potential competing interests: Tanatorn Tantipiriyakul reports financial support was provided by 10.13039/501100004508Prince of Songkla University, Phuket Campus (Project numbers COC6602073N and COC6604012S). If there are other authors, they declare that they have no known competing financial interests or personal relationships that could have appeared to influence the work reported in this paper.

## Data Availability

Data will be made available on request from the author at komsan.k@psu.ac.th.
